# Inclusion of “Toxicological Review Expiry Dates” in Art Material Labels May Further Reduce the Risk of Chronic Toxicity, Including That of Cancer

**DOI:** 10.3389/fonc.2016.00004

**Published:** 2016-01-20

**Authors:** Masood A. Shammas, Samiyah A. Rajput, Dildar Ahmad, Masood Ahmed, Zahid Mustafa, Gulzar Ahmad

**Affiliations:** ^1^Department of Adult Oncology, Harvard (Dana Farber) Cancer Institute, Boston, MA, USA; ^2^InfoTox International Inc., Riverside, CA, USA; ^3^Department of Anatomy and Histology, College of Medicine, Qassim University, Buraydah, Saudi Arabia; ^4^University of California Riverside, Riverside, CA, USA

**Keywords:** chronic toxicity, cancer, art materials, labels, LHAMA

Art materials are used by individuals of all ages, ranging from kindergarten children to adults studying the arts at a university level, as well as the general public, senior citizens, and mentally compromised individuals. Art materials have the potential of containing toxic materials, including carcinogens (such as nickel, chromium, cadmium, and lead) and reproductive toxins in their formulations. These toxic chemicals can be present as a result of intentional addition or contaminants. Such occurrences are expected to be higher in products manufactured in countries where product safety laws are less strict or not properly enforced.

Exposure to carcinogenic chemicals causes DNA damage leading to mutations that can activate oncogenes or inactivate antioncogenes, thus, predisposing cells to oncogenesis. Importantly, the heavy metals, such as nickel, can also dysregulate homologous DNA recombination (HR) (1). Although HR in normal cells is the most precise DNA repair mechanism and plays a vital role in the maintenance of genomic integrity, its dysregulation can induce genomic instability (2, 3), which is associated with progression in cancer (2). Data from our laboratory also show that exposure to nickel is associated with induction of a panel of HR genes, including (RAD51, RAD51 paralogs, RAD50, and RAD23) as well as leads to increased HR activity, genomic instability, and development of drug resistance in cancer (myeloma) cells (2).

Toxic metals (such as nickel, copper, mercury, and lead) also induce breaks in DNA. Exposure to such chemicals even at low doses can be risky because their genotoxic effect can potentially be increased by their interactions with each other and/or with other environmental or genetic factors. It has been demonstrated that simultaneous exposure to more than one heavy metals is associated with increased toxicity, relative to that observed following exposure to single metal (4). Toxicity of these compounds can also be increased in the presence of X-rays. A model proposed by Hengstler et al. (5) suggests that simultaneous exposure to multiple metals can lead to several fold increase in the toxicity relative to that induced by single compound. We, therefore, believe that constant exposure to material containing such chemicals can potentially increase cancer risk, especially in the individuals with genetic and/or other susceptibilities, including those related to certain lifestyles (6). Consistently, the reports suggest that there is an increased incidence of death due to cancers of various organs in artists (7, 8).

Art Materials that do contain toxic materials can have serious adverse health effects among users, especially small children and those with mental health issues. To minimize such effects, the US Government has enacted certain product safety laws and regulations. One of those regulations is the Federal Hazardous Substances Act (FHSA), which is being implemented by the US Consumer Product Safety Commission (CPSC). One of the components of the FHSA is “Labeling of Hazardous Art Materials Act (LHAMA).” This section deals with toxicological reviews of Art Material formulations for chronic toxicity and proper labeling.

Labeling of Hazardous Art Materials Act requires that all art materials offered for sale to consumers of all ages in the US must undergo a toxicological review by a qualified toxicologist such as Diplomate American Board of Toxicology (DABT) of the complete formulation of each product to determine the product’s potential for producing adverse chronic health effects; in addition, the art materials be properly labeled for acute and chronic hazards, as required by the LHAMA (9) and the FHSA (10), respectively.

An “art material” or “art material product” means any substance marketed or represented by the producer or repackager as suitable for use in any phase of the creation of any work of visual or graphic art of any medium and packaged in sizes intended for individual users of any age or those participating in a small group (11).

After toxicological reviews have been conducted for chronic hazards on art material formulations, the qualified toxicologists then recommend appropriate statements for art materials and also recommend a conformance statement. The conformance statement can read: “Conforms to ASTM Practice D-4236”; “Conforms to ASTM D-4236”; or “Conforms to the health requirements of ASTM D-4236.” According to the regulation at 16 C.F.R. § 1500.14(b) (8) (i) (C) (6), these toxicological reviews are valid for the period of 5 years only. The regulation states: “[T]he producer or repackager shall have a toxicologist review as necessary, *but at least every 5 years*, art material product formulation(s) and associated label(s) based upon the then-current, generally accepted, well-established scientific knowledge.”

Based on our long-term experience in the field, we have come to realize that it is impossible for the consumers to fully benefit from the 5-year re-review requirement; this is because either most consumers are unaware of 5-year re-reviewing requirement or they have no practical means to verify that the 5-year expiry period has already expired. Verification is only possible when the expiry date of the toxicological review or the date of toxicological review is marked on the Art Material label. This practice is also expected to help CPSC inspector assure compliance to 5-year re-review requirement.

In the absence of expiry dates of the toxicological reviews, it is impossible for consumers to know if the art materials they buy and use are still in compliance with the Federal regulation and are in fact safe based on the current generally accepted, well-established scientific knowledge. Therefore, we suggest that the CPSC amends the LHAMA and incorporates the requirement of adding the dates toxicological reviews are conducted or the dates toxicological reviews will expire.

This amendment will be in line with Section 103 of the CPSIA entitled “Tracking Labels for Children’s Products” (12) and Tracking Label Requirement for Children’s Products (12) that mandate, in pertinent part, “distinguishing marks” on all children’s products and their packaging to enable the manufacturer and the ultimate purchaser to “ascertain” certain source and production information. These markings are to enable the manufacturer, retailers, and the ultimate consumer to ascertain the manufacturer or private labeler, location and date of production of the product, and cohort information (batch, run number, or other identifying characteristic). These new requirements become effective on August 14, 2009.

We believe that this practice will not only remove hundreds of non-compliant Art Materials out of the US market especially those imported from other countries, but will also protect US consumers, especially children, mentally compromised individuals, and the elderly from unchecked chronically toxic chemicals, including carcinogens and reproductive toxins.

## Author Contributions

MS, SR, DA, MA, ZM, and GA equally contributed to the manuscript.

## Conflict of Interest Statement

The authors declare that the research was conducted in the absence of any commercial or financial relationships that could be construed as a potential conflict of interest.
